# The Effect of Laparoscopic Endometrioma Surgery on Anti-Müllerian Hormone: A Systematic Review of the Literature and Meta-Analysis

**DOI:** 10.5935/1518-0557.20210060

**Published:** 2022

**Authors:** José Moreno-Sepulveda, Carolina Romeral, Geraldine Niño, Assumpció Pérez-Benavente

**Affiliations:** 1 Obstetrics and Gynecology Department, Universitat Autònoma de Barcelona Campus Universitario UAB, 08193 Bellaterra, Cerdanyola del Vallès, Spain; 2 Clínica de la Mujer Medicina Reproductiva Alejandro Navarrete 2606, Viña del Mar, Chile; 3 Obstetrics and Gynecology Department, Hospital de Manacor Carretera Manacor Alcudia, s/n, 07500 Manacor, Spain; 4 Hospital Carlos Van Buren San Ignacio 725, Valparaíso, Chile; 5 Gynecologic Oncology Unit, Vall d'Hebron University Hospital, Passeig de la Vall d'Hebron, 119, 08035, Barcelona, Spain

**Keywords:** anti-Müllerian hormone, endometriosis, endometrioma, laparoscopic surgery, ovarian reserve

## Abstract

**Objective:**

This study aimed to assess the effect of endometrioma surgery on ovarian reserve by measuring anti-Müllerian hormone (AMH) levels.

**Methods:**

This systematic review and meta-analysis included observational studies and randomized clinical trials published in English referenced in MEDLINE, SCOPUS and Cochrane (1982-2019). We included studies that reported AMH levels in the pre and post-operative period of patients undergoing laparoscopic surgery for endometrioma. Preoperative AMH was defined as the baseline AMH; short term AMH was measured no later than a month after surgery; medium term AMH was measured between one and six months after surgery; and long-term AMH was measured six or more months after surgery.

**Results:**

Thirty-six studies met the inclusion criteria. A significant decrease was observed in short, medium and long-term post-operative AMH levels when compared with baseline AMH. However, there were no differences between short and long-term post-operative AMH levels, suggesting a non-significant recovery after one year of follow-up. A significant decrease in post-operative AMH was observed in bilateral endometriomas compared with unilateral cases. In addition, patients with endometriomas presented a significant decline in post-operative AMH compared with patients with other benign ovarian conditions. The decrease in post-operative AMH was significantly greater in bilateral cystectomy when compared with vaporization with bipolar energy or laser. We also observed a greater decrease in post-operative AMH with bipolar energy hemostasis compared with suture and hemostatic agents. These results should be taken with caution due to the high heterogeneity of the studies analyzed.

**Conclusions:**

Endometrioma surgery has a deleterious effect on short, medium, and long-term post-operative AMH levels. Bilateral endometriomas and endometriomas greater than 7 cm have been associated with greater decreases in AMH. The mechanical resection of healthy tissue and the inflammatory damage on the ovarian cortex might explain the diminishing of ovarian reserve.

## INTRODUCTION

Endometriosis is a benign disease characterized by the presence of endometrial tissue, both stroma and glands, outside the uterine cavity. The disease has a high prevalence, as approximately 10% of women of childbearing age might suffer from it (Hirsch *et al*., 2018). The most frequent location is the ovary, followed by the pelvic peritoneum, uterosacral ligaments, and tubes. Ovarian endometriomas are found in up to 20% of patients with endometriosis (Zondervan *et al*., 2018). Clinical presentation varies, with 20-30% of the patients remaining asymptomatic, while others may develop hypogastric pain, dysmenorrhea, dyspareunia, chronic pelvic pain, menstrual disorders and infertility (Zondervan *et al*., 2018).

Surgical techniques for endometriosis include the excision of endometriomas larger than 4 cm and adhesiolysis (Duffy *et al*., 2014). Different treatments focus specifically on endometriomas. In a Cochrane review, Hart *et al*. (2008) concluded that laparoscopic cystectomy was the most favorable technique to avoid recurrences in the treatment of endometriomas compared with drainage and ablation, since the procedure also improved pregnancy rates compared with other techniques. The most common technique used for ovarian cystectomy is decapsulation. The consequent bleeding from the remaining ovarian stroma is usually controlled with bipolar coagulation and/or suturing.

In recent years, many studies have discussed the effect of ovarian surgery on ovarian reserve and the possible risk of early ovarian failure (Duffy *et al*., 2014; Saito *et al*., 2018; Choi *et al*., 2018). Ovarian reserve is defined as the reproductive potential at a certain moment; it is determined by the quantitative analysis of the follicular count by ultrasound or biochemical tests such as measurement of serum levels of FSH, estradiol, inhibin B, and anti-Müllerian hormone (AMH) (Kline *et al*., 2005; La Marca *et al*., 2010; Committee on Gynecologic Practice, 2015). The decline of the ovarian reserve is a continuous and irreversible physiological process, with great variability at an ethnic, family or individual level (Farquhar *et al*., 2019).

Decreases in ovarian reserve after laparoscopic cystectomy have been reported, and studies have attributed it to the irreversible damage caused by bipolar electrocoagulation to achieve proper hemostasis. This damage might be secondary to the thermal effects on the ovarian stroma and vascularization, in addition to accidental and involuntary damage to healthy follicles during cyst excision (Asgari *et al*., 2016).

AMH might be the most practical ovarian reserve marker for several reasons. AMH expressed by the granulosa cells of the active developing pre-antral follicles plays an important role in the physiology of the ovary since its initial recruitment (Durlinger *et al*., 2002). This phase is still independent of gonadotropins, making it a stable hormone between and during cycles. The decrease in the number of follicles that occurs over time leads to a gradual and physiological decrease in the levels of this hormone, starting at the age of 21 (Farquhar *et al*., 2019).

To avoid follicular damage and thus preserve ovarian function, new methods are being included to control bleeding, such as suturing, plasma-jet vaporization, and coagulation with other hemostatic agents (Asgari *et al*., 2016; Kang *et al*., 2015). However, there is controversy regarding the effectiveness of such methods (Song *et al*., 2014; Sönmezer *et al*., 2013).

Recent studies have reported that endometriosis surgery has a negative impact on ovarian reserve, as shown in post-operative follow-up of AMH and antral follicles. However, there is no consensus as to whether this is due to endometriosis itself, its severity, or to factors specific to surgery, such as surgical approach and hemostatic technique (Chang *et al*., 2010; Sugita *et al*., 2013; Alborzi *et al*., 2014; Iwase *et al*., 2016; Ozaki *et al*., 2016; Goodman *et al*., 2016).

In accordance with the Patients, Intervention, Comparison and Outcomes (PICO) statements, this study aimed to assess the effects of endometrioma surgery on ovarian reserve based on AMH levels at different times before and after surgery. Additionally, we evaluated the effect of laparoscopic cystectomy on ovarian reserve according to laterality and size of the lesions, the effect of laparoscopic surgery on ovarian reserve for endometrioma vs. surgery for benign ovarian conditions, and the effect of hemostatic technique during laparoscopic surgery for endometrioma on ovarian reserve.

## MATERIAL AND METHODS

This systematic review of the literature and meta-analysis did not involve interventions in humans. Therefore, approval was not required from an Ethics Review Committee. We used the "Preferred Reporting Items for Systematic Reviews and Meta-analysis" (PRISMA) statement to report results (Shamseer *et al*., 2015).

### Search Strategy

This systematic review of the literature and meta-analysis included observational studies evaluating the effect of endometrioma surgery on ovarian reserve measured through AMH levels. Searches were performed in the following databases: MEDLINE, SCOPUS and Cochrane from 1982 to January 2019. In addition, we searched for references cited in relevant studies. The search combined terms and descriptions associated with the variants of these interventions and the study population. They included ovarian endometriosis, gynecological laparoscopy, endometriosis surgery, and AMH. The search strategy was modified to make it compatible with the syntax of each database consulted.

### Data collection and analysis

Two authors (CR and JM) independently reviewed the titles and abstracts of the studies that the search yielded. The ones that met the predefined criteria were selected for inclusion in the review. Full texts of all citations that might meet the predefined selection criteria were obtained. Disagreements and doubts were resolved by consensus of both authors. Both authors critically analyzed the results and used the GRADE System to rate the quality of the evidence and grade the strength of the recommendation for each result (Schünemann *et al*., 2013).

### Selection criteria

Inclusion criteria: The review included retrospective or prospective observational studies and randomized clinical trials that reported AMH levels in the pre and post-operative period of patients undergoing laparoscopic surgery for endometrioma. The studies included patients with benign ovarian disease and patients treated without surgery. The selection criteria are described in Table 1.

**Table 1.** Description of the studies included in the meta-analysis.

Study	Location / Years	Study design	Participants	Outcomes	Study quality (NOS)
Uncu *et al*., 2013	South Korea 2008 - 2009	Prospective cohort	27	Post-operative AMH short term *vs*. pre-operative AMH Post-operative AMH medium term *vs*. pre-operative AMH	6/9
Uncu *et al*., 2013	Turkey 2008 - 2011	Prospective cohort	60	Post-operative AMH short term *vs* . pre-operative AMH Post-operative AMH long term *vs*. pre-operative AMH Post-operative AMH short *vs*. long term	7/9
Alborzi *et al.*, 2014	Iran 2010 - 2012	Prospective cohort	193	Post-operative AMH short term *vs* . pre-operative AMH Post-operative AMH medium term *vs*. pre-operative AMH Post-operative AMH long term *vs*. pre-operative AMH Post-operative AMH short vs. long term Post-operative AMH EO unilateral *vs*. bilateral short term Post-operative AMH EO unilateral *vs*. bilateral medium term Post-operative AMH EO unilateral *vs*. bilateral long term	7/9
Tanprasertkul *et al*., 2014	Thailand 2012 - 2013	Prospective cohort	77	Post-operative AMH short term *vs* . pre-operative AMH Post-operative AMH medium term *vs*. pre-operative AMH Post-operative AMH long term *vs*. pre-operative AMH Post-operative AMH short vs. long term Post-operative AMH EO unilateral *vs*. bilateral short term Post-operative AMH EO unilateral *vs*. bilateral medium term Post-operative AMH EO unilateral *vs*. bilateral long term Post-operative AMH sealing agents *vs*. bipolar coagulation	6/9
Chen *et al*., 2014	China 2013 - 2014	Prospective cohort	98	Post-operative AMH short term *vs*. pre-operative AMH Post-operative AMH EO vs. No EO short term	8/9
Vignali *et al*., 2015	Italy 2009 - 2010	Prospective cohort	22	Post-operative AMH short term *vs*. pre-operative AMH Post-operative AMH medium term *vs*. pre-operative AMH Post-operative AMH long term *vs*. pre-operative AMH Post-operative AMH short *vs*. long term	9/9
Saito *et al.*, 2018	Japan 2011 - 2013	Prospective cohort	62	Post-operative AMH short term *vs*. pre-operative AMH Post-operative AMH long term *vs*. pre-operative AMH Post-operative AMH short *vs*. long term Post-operative AMH EO unilateral *vs*. bilateral short term Post-operative AMH laser ablation *vs*. unilateral cystectomy Post-operative AMH laser ablation *vs*. bilateral cystectomy	8/9
Muzii *et al*., 2019	Italy 2015 - 2016	Prospective cohort	52	Post-operative AMH short term *vs*. pre-operative AMH Post-operative AMH long term *vs*. pre-operative AMH Post-operative AMH short *vs*. long term Post-operative AMH EO *vs*. No EO medium term	8/9
Biacchiardi *et al*., 2011	Italy 2007 - 2009	Prospective cohort	43	Post-operative AMH long term *vs*. pre-operative AMH	7/10
Ercan *et al*., 2011	Turkey 2008 - 2010	Prospective cohort	36	Post-operative AMH medium term *vs*. pre-operative AMH	9/9
Hwu *et al*., 2011	Taiwan 2007 - 2010	Prospective cohort	1642	Post-operative AMH medium term *vs*. pre-operative AMH Post-operative AMH EO unilateral *vs*. bilateral short term	7/9
Kitajima *et al*., 2011	Japan 2007 - 2009	Prospective cohort	32	Post-operative AMH medium term *vs*. pre-operative AMH	7/9
Celik *et al*., 2012	Turkey 2009 - 2010	Prospective cohort	65	Post-operative AMH medium term *vs*. pre-operative AMH Post-operative AMH long term *vs*. pre-operative AMH	8/9
Kwon *et al*., 2014	South Korea 2011 - 2013	Prospective cohort	100	Post-operative AMH medium term *vs*. pre-operative AMH Post-operative AMH EO *vs*. No EO medium term	9/9
Salihoğlu *et al*., 2016	Turkey 2013 - 2014	Prospective case control	67	Post-operative AMH medium term *vs*. pre-operative AMH Post-operative AMH EO *vs*. No EO short term	7/9
Kim *et al*., 2017	South Korea 2011 - 2012	Prospective cohort	75	Post-operative AMH medium term *vs*. pre-operative AMH Post-operative AMH EO *vs*. No EO medium term Post-operative AMH EO unilateral *vs*. bilateral medium term	9/9
Tsolakidis *et al*., 2010	Greece 2005 - 2007	Randomized clinical trial	20	Post-operative AMH long term *vs*. pre-operative AMH Post-operative AMH laser ablation *vs*. cystectomy	9/9
Iwase *et al*., 2016	Japan 2008 - 2009	Randomized clinical trial	20	Post-operative AMH long term *vs*. pre-operative AMH Post-operative AMH laser ablation *vs*. cystectomy	9/9
Chun *et al*., 2016	Taiwan 2010 - 2013	Prospective cohort	65	Post-operative AMH EO *vs*. No EO short term	8/9
Ergun *et al*., 2015	Turkey 2011 - 2013	Prospective cohort	50	Post-operative AMH EO *vs*. No EO medium term Post-operative AMH EO unilateral *vs*. bilateral medium term Post-operative AMH EO > 7 cm *vs*. < 7 cm	7/9
Hirokawa *et al*., 2011	Japan 2008 - 2010	Prospective cohort	38	Post-operative AMH EO unilateral *vs*. bilateral short term	5/9
Saito *et al*., 2014	Japan 2011 - 2013	Prospective cohort	99	Post-operative AMH EO unilateral *vs* . bilateral short term Post-operative AMH laser ablation *vs*. unilateral cystectomy Post-operative AMH laser ablation *vs.* bilateral cystectomy	9/9
Shao *et al*., 2016	China 2012 - 2013	Prospective cohort	80	Post-operative AMH EO unilateral *vs*. bilateral long term	8/9
Mehdizadeh Kashi *et al*., 2017	Iran 2012 - 2013	Prospective cohort	70	Post-operative AMH EO unilateral *vs*. bilateral long term	8/8
Kovačević *et al*., 2018	Serbia 2013 - 2016	Prospective cohort	54	Post-operative AMH EO unilateral *vs*. bilateral long term	7/9
Ercan *et al*., 2010	Turkey 2007 - 2008	Prospective cohort	47	Post-operative AMH EO unilateral *vs*. bilateral short term	9/9
Wang *et al*., 2019	China 2014 - 2017	Prospective cohort	171	Post-operative AMH EO > 7 cm vs. < 7 cm Post-operative AMH suture *vs*. bipolar coagulation	5/8
Giampaolino *et al*., 2015	Italy 2012 - 2014	Randomized clinical trial	76	Post-operative AMH laser ablation *vs*. unilateral cystectomy	7/9
Candiani *et al*., 2018	Italy 2017 - 2018	Randomized clinical trial	60	Post-operative AMH laser ablation *vs*. cystectomy	6/9
Ferrero *et al*., 2012	Italy 2007 - 2010	Randomized clinical trial	100	Post-operative AMH suture *vs*. bipolar coagulation	8/9
Li *et al*., 2013	China 2008 - 2010	Prospective cohort	162	Post-operative AMH suture *vs*. bipolar coagulation Post-operative AMH ultrasound *vs*. bipolar coagulation	7/9
Takashima *et al*., 2013	Japan 2008 - 2010	Prospective cohort	44	Post-operative AMH suture *vs*. bipolar coagulation	8/9
Song *et al*., 2015	South Korea 2011 - 2014	Prospective cohort	125	Post-operative AMH suture *vs*. bipolar coagulation	8/9
Zhang *et al*., 2016	China 2013	Randomized clinical trial	207	Post-operative AMH suture *vs*. bipolar coagulation Post-operative AMH ultrasound *vs*. bipolar coagulation	9/9
Sönmezer *et al*., 2013	Turkey 2010	Randomized clinical trial	30	Post-operative AMH sealing agents *vs.* bipolar coagulation	9/9
Song *et al*., 2014	South Korea 2012 - 2013	Randomized clinical trial	100	Post-operative AMH sealing agents *vs*. bipolar coagulation	9/9
Choi *et al*., 2018	South Korea 2014 - 2016	Randomized clinical trial	80	Post-operative AMH sealing agents *vs*. bipolar coagulation	9/9

**Exclusion criteria:** The analysis was limited to evaluating the AMH and other studies that only considered other variables related to ovarian reserve. Reproductive results such as spontaneous pregnancy rates or assisted reproduction were excluded. Furthermore, patients undergoing laparoscopic surgery who presented other forms of endometriosis (superficial or deep) were not included.

### Outcome Measures

Outcomes were measured in terms of post-operative AMH levels in different settings. The unit of measurement of AMH level was nanograms per milliliter and SD.

Preoperative AMH was defined as the baseline AMH; short term AMH was measured no later than a month after surgery; medium term AMH was measured between one and six months after surgery; and long-term AMH was measured six or more months after surgery.

### Quality and risk of bias of included studies

Only cohort studies and randomized clinical trials were included in this systematic review and meta-analysis. Quality assessment was performed using the internationally accepted Newcastle-Ottawa scale (Stang, 2010). This scale is useful for assessing potential biases in the selection, comparability, exposure, and results stages. Table 2 shows the assessment of quality and risk of bias of the included studies.

**Table 2.** Summary of the results.

Outcome	Mean difference (95% CI)	Participants	Number of studies	Quality of evidence (GRADE)
**Post-operative versus pre-operative AMH**				
Short term	-1.62 [-2.21, -1.02]	810	8	⊕⊕⊖⊖ low
Medium term	-1.31 [-1.68, -0.93]	1376	13	⊕⊕⊖⊖ low
Long term	-1.54 [-1.89, -1.19]	932	9	⊕⊕⊖⊖ low
Short term *vs*. long term	0.11 [-0.08, 0.30]	700	6	⊕⊕⊖⊖ low
**Post-operative AMH Endometrioma vs. other benign ovarian pathology**				
Short term	-0.96 [-1.34, -0.58]	229	4	⊕⊕⊖⊖ low
Medium term	-1.40 [-2.09, -0.71]	287	4	⊕⊕⊖⊖ low
**Post-operative AMH Bilateral vs. Unilateral Endometrioma**				
Short term	-0.86 [-1.24, -0.49]	759	7	⊕⊕⊖⊖ low
Medium term	-0.86 [-1.44, -0.29]	534	4	⊕⊕⊖⊖ low
Long term	-0.75 [-1.18, -0.33]	617	5	⊕⊕⊖⊖ low
**Post-operative AMH according to size**				
Endometrioma> 7 cm *vs*. <7 cm	-0.50 [-0.65, -0.35]	197	2	⊕⊕⊕⊖ moderate
**Post-operative AMH according to surgical technique**				
Bipolar ablation *vs*. unilateral cystectomy	0.41 [-0.23, 1.04]	114	3	⊕⊕⊕⊖ moderate
Bipolar ablation *vs*. bilateral cystectomy	0.72 [0.19, 1.25]	69	2	⊕⊕⊕⊕ high
Laser ablation *vs*. cystectomy	0.86 [0.55, 1.16]	80	2	⊕⊕⊕⊖ moderate
**Post-operative AMH according to hemostatic technique**				
Suture vs. bipolar energy	0.50 [0.19, 0.81]	686	6	⊕⊕⊕⊖ moderate
Sealing agents *vs*. bipolar energy	0.53 [0.25, 0.81]	258	4	⊕⊕⊕⊕ high
Ultrasound *vs*. bipolar energy	0.00 [-0.24, 0.24]	246	2	⊕⊕⊕⊕ high

### Statistical analysis

A meta-analysis was performed using REVMAN 5.3. To determine the combined effect of each variable, we used a Mantel-Haenszel model and applied the fixed effects model. Mean difference (MD) was calculated to measure the absolute difference between the mean values in two different groups, accompanied by 95% confidence intervals (CI). Statistical significance was established at values of *p*<0.05. The degree of variation between studies attributable to heterogeneity was assessed with the I^2^ statistical test. When heterogeneity was greater than 50% (I^2^> 50%), the random effects model was applied (Higgins *et al*., 2003).

## RESULTS

Search results and characteristics of included studies

**Figure 1:**
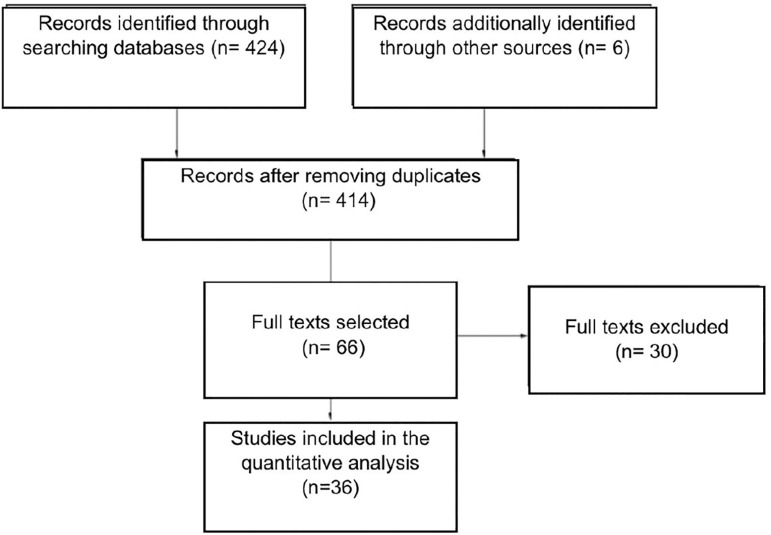
Flowchart detailing the selection of studies for inclusion in the meta-analysis. PRISMA.

The search yielded 424 articles; however, 352 were excluded in the title/abstract selection. Six articles were added from the references of the most relevant publications. One or the two reviewers considered the remaining 66 studies eligible, but exclusions were made, as explained in Figure 1. In the end, 36 studies met the inclusion criteria and were included in the study.

### Description of included studies

Of the 36 studies that met the inclusion criteria, studies with NOS scores > 7 were considered of high quality. The characteristics of the included studies are provided in Table 1.

### Summary of results

Table 2 shows a summary of the study results.

#### I. Effect of laparoscopic cystectomy of an ovarian endometrioma on ovarian reserve: post-operative AMH versus baseline AMH

#### a. Post-operative short-term AMH versus baseline AMH

Eight studies were included in the analysis, with a total of 405 patients enrolled in each group.

**Figure 2:**
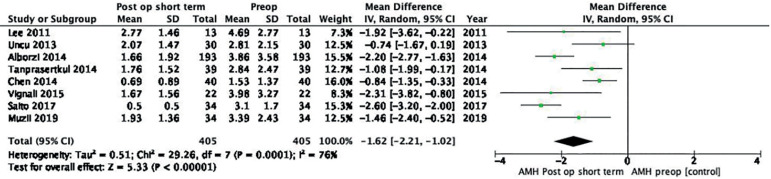
Forest plot short-term post-operative AMH *versus* baseline AMH.

Random effects analysis showed a mean difference (MD) of -1.62 (95% CI -2.21, -1-02; I^2^ = 76%) when short-term post-operative AMH and baseline AMH levels were compared (Figure 2). The quality of the evidence was low according to GRADE.

#### b. Post-operative medium-term AMH versus baseline AMH

**Figure 3:**
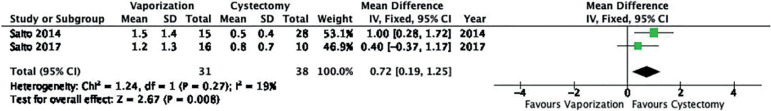
Forest plot medium-term post-operative AMH *versus* baseline AMH.

Thirteen studies with a total of 688 patients in each group were compared. Random effects analysis showed an MD of -1.31 (95% CI -1.68, -0.93; I^2^ = 94%) when post-operative medium-term AMH and baseline AMH levels were compared (Figure 3). The quality of the evidence was low according to GRADE.

#### c. Long-term post-operative AMH versus baseline AMH

**Figure 4:**
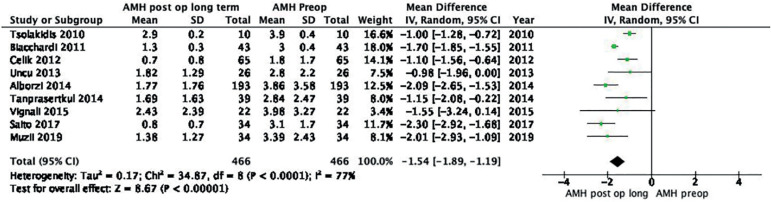
Forest plot long-term post-operative AMH *versus* baseline AMH.

Random effects analysis of nine studies including 466 patients in each group showed an MD of -1.54 (95% CI -1.89, -1.19; I^2^ = 77%) when long-term post-operative AMH and baseline AMH were compared (Figure 4). The quality of the evidence was low according to GRADE.

#### d. Long-term post-operative AMH versus short-term post-operative AMH

**Figure 5:**
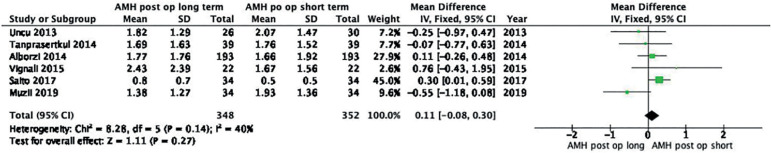
Forest plot long-term post-operative AMH *versus* short-term post-operative AMH.

The analysis of six studies with 348 patients in the long-term post-operative AMH group and 352 in the short-term AMH group demonstrated an MD of 0.11 (95% CI -0.08, 0.30; I^2^ = 40%) when long-term post-operative AMH and short-term post-operative AMH were compared via fixed effects analysis (Figure 5). The quality of the evidence was low according to GRADE.

#### II. Effect of laparoscopic cystectomy on ovarian reserve according to laterality of the lesions

#### a. Short-term AMH in unilateral versus bilateral endometriomas

**Figure 6:**
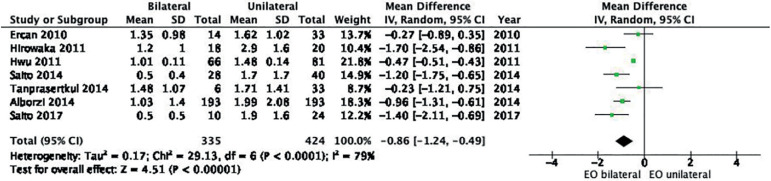
Forest plot AMH short term in unilateral versus bilateral endometriomas.

Seven studies were included in the analysis, with a total of 355 patients included in the group with bilateral endometriomas and 424 in the group with unilateral endometriomas. Random effects analysis showed lower post-operative AMH levels in patients with bilateral endometriomas compared with subjects with unilateral endometriomas in the short-term (MD -0.86, 95% CI -1.24, -0.49; I^2^ = 79%, Figure 6). The quality of the evidence was low according to GRADE.

#### b. Medium-term AMH in unilateral versus bilateral endometriomas

**Figure 7:**
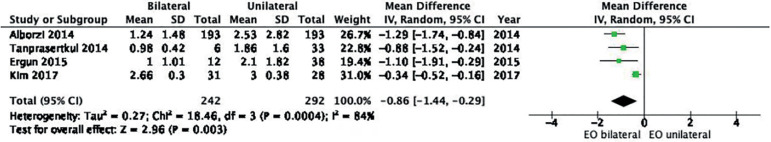
Forest plot AMH in the medium term in unilateral *versus* bilateral endometriomas.

The analysis of four studies with a total of 242 patients with bilateral endometriomas and 292 with unilateral endometriomas revealed lower post-operative AMH levels in patients with bilateral endometriomas compared with patients with unilateral endometriomas in the medium-term (MD of -0.86, 95% CI -1.44, -0.29; I^2^ = 84%, Figure 7). The quality of the evidence was low according to GRADE.

#### c. Long-term AMH in unilateral versus bilateral endometriomas

**Figure 8:**
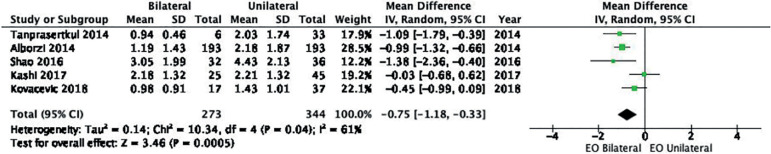
Forest plot long-term AMH in unilateral *versus* bilateral endometriomas.

Five studies were included in the analysis, with a total of 273 patients with bilateral endometriomas and 344 with unilateral endometriomas. Random effects analysis showed lower post-operative AMH levels in patients with bilateral endometriomas compared with subjects with unilateral endometriomas in the long-term (MD of -0.75, 95% CI -1.18, -0.33; I^2^ = 61%, Figure 8). The quality of the evidence was low according to GRADE.

#### III. Effect of laparoscopic surgery for endometrioma on ovarian reserve compared with benign ovarian conditions

#### a. Post-operative short-term AMH in subjects with endometrioma versus benign ovarian disease

**Figure 9:**
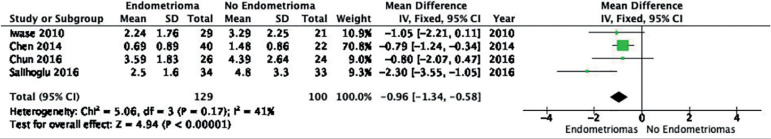
Forest plot short-term post-operative AMH in endometriomas *versus* other benign ovarian pathology.

The four studies included in the analysis reported lower post-operative AMH levels in patients with endometriomas versus subjects without endometriomas in the short-term (MD of -0.96, 95% CI -1.34, -0.58; I^2^ = 41%, Figure 9). Fixed effect analysis included 129 patients in the endometrioma group and 100 in the non-endometrioma group. The quality of the evidence was low according to GRADE.

#### b. Post-operative medium-term AMH in subjects with endometrioma versus benign ovarian disease

**Figure 10:**
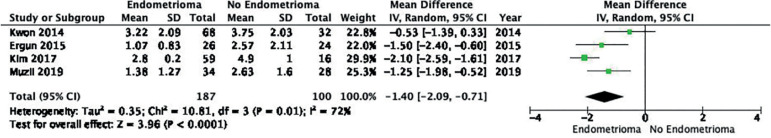
Forest plot AMH post-operative medium term in endometriomas *versus* benign ovarian pathology.

Random effects analysis comparing four studies with a total of 187 patients in the endometrioma group and 100 in the non-endometrioma group showed lower post-operative AMH levels in patients with endometriomas in the medium-term (MD of -1.40, 95% CI -2.09, -0.71; I^2^ = 72%, Figure 10). The quality of the evidence was low according to GRADE.

#### IV. Effect of laparoscopic cystectomy on ovarian reserve according to lesion size

#### a. Post-operative AMH in endometriomas greater than 7 cm *versus* smaller than 7 cm

**Figure 11:**
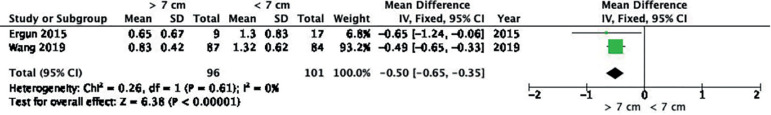
Forest plot AMH post-operative in endometriomas greater than 7 cm versus less than 7 cm.

The comparison of two studies with a total of 96 patients included in the group of endometriomas greater than 7 cm and 101 in the group of endometriomas measuring less than 7 cm presented evidence of moderate quality according to GRADE. Fixed effects analysis showed lower post-operative AMH levels in patients with endometriomas greater than 7 cm in the short term (DM of -0.50, 95% CI -0.65, -0.35; I^2^ = 0%, Figure 11).

#### V. Effect of laparoscopic surgical technique on ovarian reserve in patients with endometriomas

#### a. Post-operative AMH after vaporization with bipolar energy *versus* unilateral cystectomy

**Figure 12:**
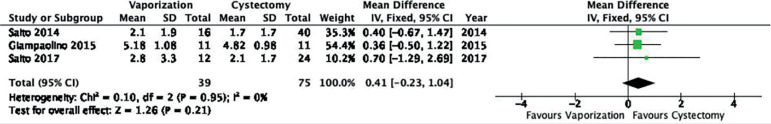
Forest plot AMH post-operative post vaporization with bipolar energy *versus* unilateral cystectomy.

Three studies were included in the analysis, with a total of 39 patients included in the group treated with bipolar energy vaporization and 75 in the unilateral cystectomy group. Fixed effects analysis showed no significant differences between means when comparing AMH levels post-vaporization with bipolar energy versus unilateral cystectomy (MD of 0.41, 95% CI -0.23, 1.04; I^2^ = 0%, Figure 12). The quality of the evidence was moderate according to GRADE.

#### b. Post-operative AMH after vaporization with bipolar energy *versus* bilateral cystectomy

**Figure 13:**
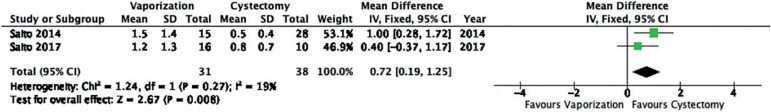
Forest plot AMH post-operative post vaporization with bipolar energy *versus* bilateral cystectomy.

The analysis of two studies with only 31 patients in the group treated with bipolar energy vaporization and 38 in the group treated with bilateral cystectomy showed higher post-operative AMH levels after post-vaporization with bipolar energy (DM of 0.72, 95% CI 0.19, 1.25; I^2^ = 19%, Figure 13). The quality of the evidence was high according to GRADE.

#### c. Post-operative AMH after laser vaporization *versus* cystectomy

**Figure 14:**
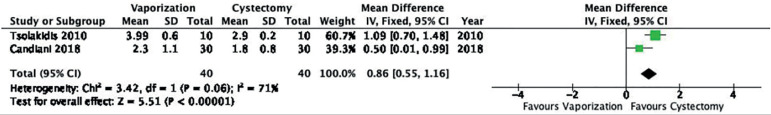
Forest plot AMH post-operative post laser vaporization *versus* cystectomy.

The analysis of 40 patients included in each group from two studies revealed higher post-operative AMH levels after laser vaporization versus cystectomy (MD of 0.86, 95% CI 0.55, 1.16; I^2^ = 71%, Figure 14). The quality of the evidence was moderate according to GRADE.

#### VI. Effect of hemostatic technique during laparoscopic endometrioma surgery on ovarian reserve

#### a. Post-operative AMH based on sutured hemostasis versus bipolar energy hemostasis

**Figure 15:**
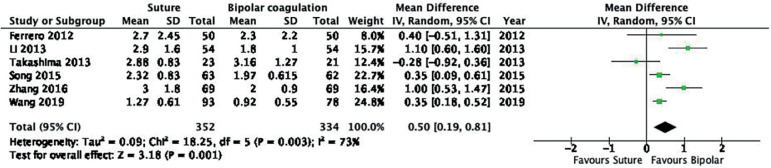
Forest plot AMH post-operative according to hemostasis with suture *versus* hemostasis with bipolar energy.

Six studies were included in the analysis, with a total of 352 patients treated with sutured hemostasis and 344 with bipolar energy hemostasis. Random effects analysis showed higher post-operative AMH levels after sutured hemostasis (MD of 0.50, 95% CI 0.19, 1.81; I^2^ = 73%, Figure 15). The quality of the evidence was moderate according to GRADE.

#### b. Post-operative AMH according to hemostasis with hemostatic agents versus hemostasis with bipolar energy

**Figure 16:**
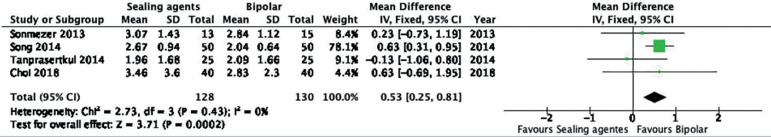
Forest plot AMH post-operative according to hemostasis treatment with hemostatic agentes versus hemostasis with bipolar energy.

Four studies including a total of 128 patients treated with hemostatic agents and 130 with bipolar energy hemostasis revealed higher post-operative AMH levels after use of hemostatic agents according to fixed effects analysis (MD of 0.53, 95% CI 0.25, 0.81; I^2^ = 0%, Figure 16). The quality of the evidence was high according to GRADE.

#### c. Post-operative AMH according to hemostasis with ultrasound versus hemostasis with bipolar energy

**Figure 17:**
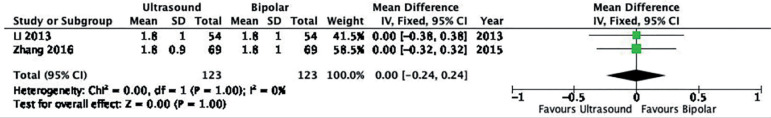
Forest plot AMH post-operative according to hemostasis with ultrasound versus hemostasis with bipolar energy.

Two studies were included in the analysis, with a total of 123 patients in each group. Fixed effects analysis showed no significant differences between means when comparing post-operative AMH levels of patients offered hemostasis with ultrasound versus hemostasis with bipolar energy (MD of 0.00 (95% CI -0.24, 0.24; I^2^ = 0%, Figure 17). The quality of the evidence was high according to GRADE.

## DISCUSSION

### Main results

In this study, we systematically reviewed the effect of laparoscopic surgical treatment of endometriomas on ovarian reserve reported in 36 studies, including a total of 4374 patients. The results showed a significant decrease in short, medium and long-term post-operative AMH when compared with baseline AMH. However, there were no differences between short and long-term post-operative AMH levels, suggesting a non-significant recovery of AMH levels after one year of follow-up. Moreover, when the effect of laparoscopic cystectomy on ovarian reserve according to the laterality of the lesions was studied, a significant decrease in post-operative AMH was observed in the short, medium and long-term in bilateral endometriomas compared with unilateral endometriomas.

When evaluating the post-operative ovarian reserve of patients with endometriomas compared with other benign ovarian conditions, the decrease in AMH was significantly more pronounced in endometriomas, both in the short and medium term. In turn, the decrease in AMH was significantly greater when the size of the endometriomas exceeded 7 cm.

In relation to the effect of laparoscopic surgery on the ovarian reserve of patients with endometriomas, post-operative AMH levels were significantly lower after bilateral cystectomy when compared with vaporization with bipolar energy. Lower post-operative AMH levels were also observed after laser vaporization when compared with cystectomy. No difference was observed when comparing post-operative AMH of patients submitted to unilateral cystectomy versus bipolar energy vaporization.

Post-operative AMH also decreased significantly with bipolar energy hemostasis compared with suture and hemostatic agents. On the other hand, this difference was not significant when compared with hemostasis with ultrasound.

### Strengths

Our study analyzed a large number of patients compared with previous reviews (Somigliana *et al*., 2012; Younis *et al*., 2019) because we included the results from several recent studies. Furthermore, a large proportion of the included studies were randomized clinical trials with good methodological quality. The included studies also stand out for having adjusted for important confounding variables such as surgical indication, fertility status, operating time, days of hospitalization, etc. These factors significantly increased the validity of our findings.

### Limitations

Several studies were excluded due to language barriers and presentation of partial results or findings that did not match our inclusion criteria. However, it was not possible to contact all authors in order to gain access to their databases. Several retrospective observational studies were included, and it was not possible to know what the AMH measurement method was.

Serum levels are not altered with prior use of hormonal contraceptives or other drugs, although decreases have been observed during the administration of anovulatory drugs or GnRH analogues, an effect that must be considered, but which seems to disappear after withdrawal of these treatments.

A disadvantage of AMH is that there are several assay types for detection, making it difficult to compare levels obtained in different studies with different methods. In practice, the serum AMH level of a patient should be interpreted evaluating the method used and its cut-off levels (La Marca *et al*., 2010).

In addition, the meta-analysis showed some highly heterogeneous values through I2 for several of the primary outcomes, suggesting that the precision of the meta-analysis is of moderate quality for these results.

### Comparison with other studies

#### Evolution of post-operative AMH

The decrease in ovarian reserve after endometrioma cystectomy has been described in numerous studies. However, these studies are highly heterogeneous and include different surgical techniques, follow-up periods, and measures to quantify the ovarian reserve.

In recent years, several authors have reported decreases in ovarian reserve sided by decreases in AMH after ovarian cystectomy (Chang *et al*., 2010; Ozaki *et al*., 2016). Nevertheless, some reports have observed a partial recovery of AMH in some women after three to 12 months of endometrioma removal (Sugita *et al*., 2013; Alborzi *et al*., 2014).

A prospective study followed up post-operative AMH levels at six and 12 months, observing a recovery of 36.4% and 72.2%, respectively, compared with baseline AMH (Vignali *et al*., 2015). Consistent with these studies, a recent systematic review reported a partial reversal of decreased post-operative AMH in the medium term compared with the short term, regardless of bilateralism (Younis *et al*., 2019). Our study, however, found no difference between post-operative AMH levels comparing the short with the long-term, after a follow-up of up to six months.

Two authors with similar findings found no differences, regardless of the surgical technique used (Chang *et al*., 2010; Ferrero *et al*., 2012). Our results should be evaluated with caution, in part due to the high heterogeneity observed during the analysis, as we wait for new studies reporting longer-term results.

In addition, some studies present limitations due to the use of combined contraceptives, since recent data indicate that they might decrease AMH levels temporarily (Kallio *et al*., 2013). However, it has been shown that a small portion of the patients included in each study group was on oral contraceptives patients, and that no difference was found when these patients were excluded from the analysis.

#### Evolution of post-operative AMH according to laterality

Our findings also indicated that greater decreases in post-operative AMH levels are observed after the resection of bilateral endometriomas compared with unilateral ones, as described in some studies (Hirokawa *et al*., 2011; Kim *et al*., 2017; Mehdizadeh Kashi *et al*., 2017) and refuted in others (Alborzi *et al*., 2014; Tanprasertkul *et al*., 2014; Ding *et al*., 2015a). However, these results are questionable due to the small number of patients studied, the loss of patients during follow-up, and the fact that surgical techniques were not evaluated.

A recent systematic review reported maximum decreases in post-operative AMH of 39.5% and 57.0% in individuals with unilateral and bilateral endometriomas, respectively (Younis *et al*., 2019).

#### Evolution of post-operative AMH according to the nature of the injury

Ovarian endometriomas per se might damage the ovarian reserve and cystectomy in patients with endometriomas cause extra damage to the ovarian reserve compared with other benign cysts (Muzii *et al*., 2019). This study observed that the decreases in post-operative AMH levels were greater in patients with endometriomas compared with benign ovarian cysts. However, a recent meta-analysis reported a similar magnitude of reduction of post-operative AMH for both groups of around 38% (Mohamed *et al*., 2016).

#### Evolution of post-operative AMH according to the size of the endometrioma

In agreement with our results, some studies have shown differences in post-operative AMH according to the size of endometriomas, with a more pronounced decrease observed in endometriomas measuring more than 5 cm in diameter (Wang *et al*., 2019). Conversely, other studies found no relationship between the diameter of the endometrioma and the percentage decrease in post-operative AMH. Many such studies enrolled small populations and took the laterality of the lesions into account in their analyses (Hirokawa *et al*., 2011; Celik *et al*., 2012; Uncu *et al*., 2013).

#### Evolution of post-operative AMH according to the surgical technique used

To date, there is no consensus on the effect of endometrioma decapsulation per se on ovarian reserve. A randomized clinical trial revealed that ablation of the cyst wall by vaporization with bipolar energy might be associated with better preservation of the ovarian reserve than cystectomy (Var *et al*., 2011). Another study revealed that ablation with plasma energy, in spite of producing similar decreases in post-operative AMH levels in the short term compared with cystectomy, yields a more significant long-term recovery (Roman *et al*., 2011).

In line with the above results, we agree that ablation by vaporization with both bipolar and laser energy is the technique of choice over cystectomy in patients wishing to preserve ovarian reserve for reproductive purposes.

#### Evolution of post-operative AMH according to the hemostatic technique used

The most used hemostatic techniques in endometrioma surgery are based on laser energy, bipolar energy or suture. However, a randomized clinical trial reported that post-operative AMH decreases significantly regardless of hemostatic method (Ferrero *et al*., 2012).

This has prompted other researchers to develop new methods to prevent ovarian damage. In a recent prospective study, bipolar coagulation was compared with suture, with the latter leading to better results in terms of preservation of the ovarian reserve (Song *et al*., 2015). The advantage of hemostatic suture includes lower cost and less biochemical complications compared with hemostatic sealants. Nonetheless, it might cause ischemic damage and adhesions.

Another study did not find a significant decrease in post-operative AMH when using hemostatic suture techniques, suggesting that a good surgical technique, without the use of bipolar energy, does not necessarily imply a reduction in ovarian reserve (Litta *et al*., 2013).

However, other studies found no significant differences in terms of changes in the post-operative ovarian reserve with either of the two hemostatic methods (Tanprasertkul *et al*., 2014; Takashima *et al*., 2013). Variations in surgical technique, surgeon experience or the different methods to measure AMH levels might explain the differences in the results described in the literature.

Another study compared the effect of hemostatic sealants and bipolar coagulation on post-operative ovarian reserve, concluding that short-term damage was more severe with bipolar coagulation. It was also reported that there were no significant differences in AMH levels between the two groups. Moreover, the levels of AMH were equal to the levels seen three months after surgery (Sönmezer *et al*., 2013). However, the sample size of this study was very small, limiting the conclusions derived from its results. A subsequent study was the first to show statistically significant differences in terms of less post-operative decrease in AMH at three months using hemostatic sealants compared with bipolar energy (Choi *et al*., 2018).

According to the results obtained in our study, three systematic reviews with meta-analysis have suggested that the use of bipolar energy is associated with greater decreases in AMH compared with non-thermal hemostatic techniques, including suturing and sealing agents (Ding *et al*., 2015b; Ata *et al*., 2015; Deckers *et al*., 2018). In any case, our study has a larger sample size, which increases the precision of the results. Long-term follow-up studies are needed, as most only assess post-operative AMH in the medium term.

### Interpretation of the Results

Many hypotheses have been formulated to explain the relationship between endometrioma excision and AMH reduction. Several authors demonstrated that the removal of ovarian endometriomas is usually more difficult due to the absence of a clear cleavage plane between the cyst capsule and the healthy ovarian parenchyma. This might cause accidental mechanical damage to the ovarian cortex causing loss of follicles and decreased ovarian reserve. On the other hand, the electrical damage of the hemostatic method used might cause microvascular and inflammatory damage to the residual ovarian tissue leading to acute damage, which might be recovered in the long-term due to revascularization and the reduction of inflammation. The relative recovery of AMH levels expressed in some articles (Sugita *et al*., 2013; Goodman *et al*., 2016) might also be attributed to a phenomenon of restructuring and growth of more primordial follicles mediated by angiogenic and inflammatory factors, especially when surgery is performed meticulously, avoiding the removal of healthy ovarian tissue. This data cannot be confirmed with our results, but long-term monitoring of the ovarian reserve of the patients might be helpful.

Regarding cyst laterality, the differences in AMH decrease after unilateral versus bilateral cystectomy might be attributed to the fact that a larger surgical procedure, such as the one associated with bilateral endometriomas, might imply, on the one hand, a greater use of suture and hemostasis, and on the other, an eventual removal of a greater proportion of healthy ovarian tissue (Alammari *et al*., 2017).

Cystectomy of endometriomas generate an apparently greater impact on ovarian reserve compared with cystectomy of other benign ovarian conditions, although it is difficult to determine whether the differences in the magnitude of the decrease in AMH levels are inherent to endometriosis or to the influence of surgical techniques (Alammari *et al*., 2017). One study postulated that endometrioma surgery led to greater damage to residual ovarian tissue compared with other types of cystectomies (Kitajima *et al*., 2011). Furthermore, theoretically the risk of accidental excision of healthy ovarian tissue is greater in endometrioma due to its inflammatory nature and the adhesions that the disease causes. A study found ovarian stroma in 80.3% of endometrioma samples, compare to only 17.2% of dermoid cyst samples (Muzii *et al*., 2019). Another study concluded that the ovarian volume was significantly lower 36 months after surgery for endometrioma than after dermoid cyst surgery (Exacoustos *et al*., 2004).

When the impact of the size of endometriomas was compared, it has been found that the ovarian cortex of endometriomas larger than 4 cm might have fibrosis and stromal loss, leading to significantly lower follicular density, compared with the contralateral ovary. In addition, the larger the cyst to be removed, the greater the amount of healthy ovarian tissue removed, and the greater the loss of ovarian reserve (Goodman *et al*., 2016; Wang *et al*., 2019). This indicates that the larger the diameter of the cyst, the greater the damage to the ovarian reserve, although this must be confirmed with further research.

#### Surgical technique and hemostasis

There are two possible mechanisms by which ovarian damage might occur during endometrioma surgery: mechanical damage and electrical damage to growing follicles. On the one hand, our results indicate that cystectomy is associated with a greater impact on ovarian reserve compared with ablation by different means. The mechanical damage and the resection of healthy tissue might explain the consequent loss of follicles (Wang *et al*., 2019).

However, it has been proposed that different hemostatic techniques used during cyst removal might compromise the vascularization of the remaining ovarian tissue and cause inflammation (Goodman *et al*., 2016). This might explain the recovery of AMH levels described in different articles (Vignali *et al*., 2015; Younis *et al*., 2019), since tissue revascularization and decreased inflammation might cause the improvement of the ovarian reserve. Although this finding could not be demonstrated in our study, possibly longer monitoring of AMH levels in operated patients might modify the results.

### Implications of the study and future directions

Endometriomas are frequent findings in clinical practice and often require surgical treatment. Our results indicate that laparoscopic surgery negatively affects the ovarian reserve, and that bilateralism and size are compounding adverse factors. However, other factors that were not evaluated, such as surgeon experience and the influence of different methods to measure AMH, might also affect the decrease in ovarian reserve after surgery.

Furthermore, it is important to determine what constitutes optimal endometrioma management based on patient subtypes, and assess measures such as conservative management (ablation). Although it has been associated with less involvement of the ovarian reserve compared with cystectomy, it is ineffective in terms of gestation rates and exposes the patient to a higher risk of recurrence.

Based on our results, we conclude that patients should be advised and informed of all aspects related to different surgical techniques and both the potential risks of decreased ovarian reserve and possible recurrence tied to each different surgical technique. Special consideration must be given to infertile patients before undergoing IVF treatment. Importantly, not all studies showed that low AMH is associated with low pregnancy rates in patients undergoing IVF.

Therefore, surgical planning must be individualized according to patient symptoms and reproductive objectives; preference should be given to conservative methods if the patient wishes to preserve her fertility.

Taking into account that the size of endometrioma is associated with significant post-operative decreases in AMH levels and that endometriosis is a progressive disease, early detection and conservative management might prevent future reproductive problems. Patients with severe endometriosis and older patients wishing to get pregnant should be informed that surgery might cause irreversible damage to the ovaries (Lessey *et al*., 2018).

#### Future directions

In order to lessen the impact of surgery on AMH, new surgical techniques are under development. One of them is sclerotherapy guided by transvaginal ultrasound. In this technique, different substances are injected into the endometrioma in order to shrink it and make it lose its pathogenic potential (Gordts & Campo, 2019). Tetracycline, methotrexate and ethanol are among the sclerosing agents being studied, with promising results (Cohen *et al*., 2017). Local alternatives are also in development of the hormonal type in order to reduce the risks of surgery (Benagiano *et al*., 2016).

New techniques such as orthotopic transplantation of ovarian tissue or cryopreservation of ovarian tissue should be considered in patients with worse reproductive prognoses and individuals requiring surgery to manage their symptoms (Donnez *et al*., 2004; 2005). Researchers in this area should guide their efforts to develop randomized clinical trials with good methodological quality in order to obtain better quality of evidence. New clinical trials with long-term monitoring of ovarian reserve are required to define the impact of different endometrioma management strategies on AMH levels.

## CONCLUSIONS

Laparoscopic resection of endometriomas produces deleterious effects on short, medium, and long-term post-operative AMH levels.

There are no differences in the decrease of post-operative AMH in the short and long-term; however, these results should be taken with caution due to the high heterogeneity of the studies included in the analysis.

Bilateral endometriomas and endometriomas greater than 7 cm are associated with a greater decrease in AMH levels compared with unilateral endometriomas and endometriomas measuring less than 7 cm.

Laparoscopic resection of endometriomas produces greater deleterious effects on post-operative AMH levels compared with other benign ovarian conditions.

Ovarian cystectomy is associated with greater post-operative AMH involvement than bipolar energy ablation in bilateral endometriomas and laser energy ablation in all endometriomas. When hemostatic techniques are compared, bipolar energy produces a greater decrease in post-operative AMH levels than suture and sealing agents.
